# Innovative Method for Determining the Toxicometric Index of Polymer Materials with the Example of Polyurethane and Polyvinyl Chloride

**DOI:** 10.3390/polym17040467

**Published:** 2025-02-11

**Authors:** Arkadiusz Głowacki, Przemysław Rybiński

**Affiliations:** Institute of Chemistry, The Jan Kochanowski University, Uniwersytecka 7, 25-406 Kielce, Poland

**Keywords:** toxicometric index, toxicity, polyurethane PUR, polyvinyl chloride PVC, gas emission, TG-FTIR, gas analysis

## Abstract

The aim of this study was to optimize a method for qualitative and quantitative determination of gaseous degradation products formed in the process of thermal decomposition in the sample. The toxicometric index was determined with the use of the coupled TG-FTIR technique (gas analyzer). The polyurethane (PUR) and polyvinyl chloride (PVC) were used for analytical studies. Based on the obtained results, it was concluded that the sample mass used for analysis, as well as the spectral range of the IR spectrum, has a crucial role in the qualitative and quantitative assessment of gaseous toxic degradation products generated during the thermal decomposition of polymeric materials. Using a gas analyzer, proprietary toxicity indices were developed, i.e., the partial toxicity index (IT_PC_) and total toxicity index (IT_GC_). It should be noted that the determined toxicity indices refer to a test sample not exceeding 10 mg. The small mass of the sample subjected to analysis allows for high resolution and repeatability of the results reading. The results of this study provide a significant methodological contribution to both the identification of gaseous degradation products formed during the thermal decomposition of materials and their quantitative detection.

## 1. Introduction

Polymeric materials are ubiquitous, constituting an integral part of daily life. This group of synthetic materials includes, among others, the following: polyethylene (PE) [1], polypropylene (PP) [2], polystyrene (PS) [3], polyamide (PA) [4], polycarbonate (PC) [5], polyurethane (PUR) [6], polyvinyl chloride (PVC) [7], acrylic plastics (PMMA) [8], and diene copolymers, such as synthetic rubber (SBR) [9,10]. Natural polymers, on the other hand, include cellulose [11], natural rubber [12], starch [13], and chitin [14].

The development of new technologies and innovations in the field of polymers is a crucial factor of progress in numerous industrial sectors [15,16]. Both the global economy and everyday activities heavily rely on the use of polymers and polymer composites. However, most polymeric materials, especially synthetic ones, represent a significant fire hazard. The term “fire hazard” encompasses not only the emission of heat but also the release of smoke and toxic gases [17,18,19,20].

Smoke emission is a significant part of the fire hazard caused by the decomposition process of polymer composites. Smoke is defined as an aerosol of solid or liquid particles generated during the processes of pyrolysis or thermo-oxidative decomposition. The extent of smoke emission depends on various factors, including the chemical composition of the material, oxygen availability, intensity of heat flux, and combustion conditions (flaming or non-flaming combustion) [21,22,23,24].

The amount of smoke generated during the thermal decomposition of a polymer is primarily determined by the chemical structure of the macromolecule. For example, PMMA undergoes thermal decomposition via depolymerization to over 90% methyl methacrylate (an oxygen-rich compound), which decomposes almost without smoke emission [25,26,27].

In contrast, PVC combustion releases large amounts of black, toxic smoke. In the initial stage of PVC decomposition (200–300 °C), significant amounts of hydrogen chloride are released and unsaturated carbon structures are formed. These structures undergo cyclization and aromatization in the subsequent thermal decomposition processes. It is well known that aromatic degradation products, when condensing in the gas phase within the flame into polyaromatic compounds, serve as excellent precursors for soot formation. Consequently, they increase the amount of emitted smoke [28,29].

**Figure 1:**
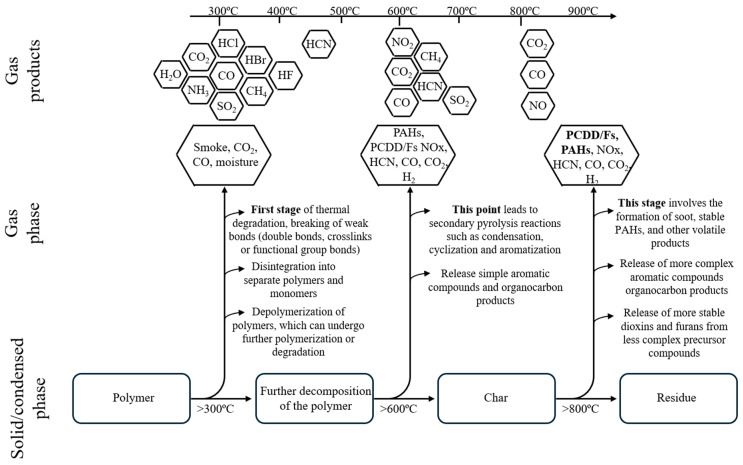
Diagram of products released during the thermal degradation of polymers.

Another example of a highly flammable material characterized by high heat release and smoke production is PUR. The thermal decomposition of PUR is a complex process during which the polymer chain breaks down into isocyanates, alcohols, amines, and carbon dioxide and monoxide. The smoke production during PUR decomposition peaks within the first 10 min after the fire outbreak [30,31,32,33,34]. The smoke produced during the thermal decomposition of polymers contains many toxic combustion products (Figure 1).

The gaseous products that pose threats to health and safety, most commonly described in scientific literature, standards, and by international institutions such as the National Institute for Occupational Safety and Health (NIOSH) [35], the National Institute of Standards and Technology (NIST) [36], the Occupational Safety and Health Administration (OSHA) [37], and the National Fire Protection Association (NFPA) [38], include the following: carbon monoxide (CO), hydrogen cyanide (HCN), hydrogen chloride (HCl), hydrogen bromide (HBr), hydrogen fluoride (HF), nitrogen oxides (NOx), and sulfur dioxide (SO_2_). These gases are frequently mentioned in scientific publications, standards, and journals. However, this does not mean that this group alone constitutes the only or primary threat to humans and the environment. Significant gaseous degradation products also include ammonia (NH_3_), polycyclic aromatic hydrocarbons (PAHs) along with their nitro derivatives, and polychlorinated dibenzodioxins and furans (PCDD/Fs). The assessment of the toxicity of gaseous degradation products emitted during the thermal decomposition and combustion of polymeric materials is based on measuring the emissions of gases such as CO, CO_2_, HCN, HCl, HBr, HF, NOx, and SO_2_.

The lethal concentration limit (LC_50_) of individual gaseous components (CO, CO_2_, HCN, HCl, HBr, HF, NOx, SO_2_) represents the concentration causing the death of 50% of a tested animal population. These threshold concentrations are used to calculate toxicometric indices. It should be noted that LC_50_ values and the range of toxicometric indices may depend on the measurement method, country, or regulatory standards.

One of the earliest standards concerning the determination of toxicometric indicators using a quartz tube furnace is ISO 02855 [39] from 1988. This standard allows for the calculation of the WLC_50_ index by determining the cumulative indicators of individual decomposition and combustion products (WLC_50M_) and then the toxicometric index (WLC_50SM_), representing the arithmetic mean of indicators at temperatures of 450 °C, 550 °C, and 750 °C. More recent standards for measuring both smoke and toxicity include the single-chamber test method for assessing smoke optical density, ISO 5659-2 [40] (Annex C), and the PN-EN 45545-2 [41] standard on fire protection in rail vehicles. The EN 45545-2 standard defines toxicity using the toxicity index CIT_G_. In this method, gases for analysis are collected twice over 30 s intervals (225–255 s and 465–495 s) and then quantified and identified using an FTIR analyzer. Recorded spectra at 240 s and 480 s allows for the potential determination of the maximum gas emissions released during the decomposition and combustion of the sample.

**Figure 2:**
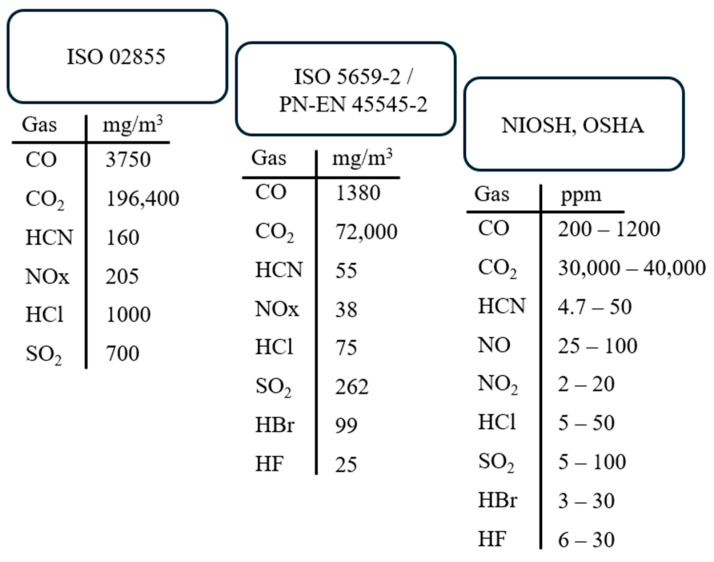
Gas concentrations according to standards ISO 02855, ISO 5659-2, PN-EN 45545-2, and organizations NIOSH 2024 and OSHA 2024.

The toxicity of gaseous degradation products is also assessed by institutions such as NIOSH (National Institute for Occupational Safety and Health) and OSHA (Occupational Safety and Health Administration). These institutions define toxicometric indicators and LC_50_ values for specific toxic gases in the context of occupational environments (Figure 2).

Differences in LC_50_ threshold values arise from the importance of hazards and the specific environments in which toxic degradation products are emitted (Figure 2). Locations with an increased risk of fire hazards require stricter limits.

In addition to the presented standards for assessing fire hazards and toxicity using methods such as the quartz tube furnace or the smoke optical density chamber coupled with an FTIR spectrophotometer, the scientific literature describes the application of other advanced analytical techniques. These include TG-FTIR, TG-MS, TG-FTIR-GC, GC-MS, GC-MS/MS, and cone calorimetry, which enable precise identification and quantitative determination of gaseous degradation products.

The aim of this study is to present a modern technique for evaluating the toxicity of polymeric materials during their thermal decomposition. The article describes the practical application of the Omega 5 gas analyzer, the process of selecting measurement conditions, and the quantitative and qualitative identification of gases generated during the thermal decomposition of polymeric materials, specifically PUR and PVC.

## 2. Methodology

### 2.1. Materials

The materials used in the study included flexible PUR foams and PVC. The PUR foam was obtained via a one-step synthesis process, using an isocyanate and a polyol in a molar ratio of NCO:OH equal to 2:1. The second material employed for comparative analyses was PVC. The PVC used was a commercial artificial leather material based on polyvinyl chloride (Polanvil, Anwil S.A. Wrocławek, Poland).

Before the experiments, the tested polymeric materials were exposed to a conditioning process under controlled conditions, specifically at a temperature of 23 ± 2 °C and relative humidity not exceeding 50 ± 5%, in accordance with the requirements of ISO 291 [42]. The conditioning process was successfully completed if, after 24 h, the sample mass did not change by more than 0.1 g or 0.1% of the initial mass.

After conditioning, the composite samples were subjected to further analyses in accordance with the planned experimental procedures.

### 2.2. Methods

The toxicity analysis of gaseous products was conducted with the use of a coupled system with a Netzsch TG 209 F1 Libra (Selb, Germany) thermogravimetric analyzer (TG) and an Omega 5 gas analyzer equipped with an FTIR spectrophotometer (Bruker Omega 5, Billerica, MA, USA). The thermal decomposition process in the thermogravimetric analyzer was carried out in the temperature range of 30–650 °C, at a constant heating rate of 10 °C/min, in an oxygen/nitrogen atmosphere with respective flow rates of 20/40 mL/min.

Samples with masses of 5, 10, and 15 mg (±1 mg) were used for the analysis. The FTIR spectrophotometer in the Omega 5 gas analyzer recorded data at intervals of 7–8 s, ensuring precise real-time monitoring of changes in gas composition. Infrared (IR) spectra were obtained using 10 scans, allowing for the accurate identification and analysis of emitted gases during the thermal decomposition of the tested materials. The IR spectra obtained with the Omega 5 gas analyzer were converted to concentrations in ppm units using OPUS GA software (version 8.7.14, Billerica, MA, USA). During the thermal decomposition process, real-time concentrations of gases such as CO, CO_2_, HCN, HCl, NH_3_, NO, and NO_2_ were recorded.

## 3. Results and Discussion

### 3.1. Selection of Sample Mass and Spectral Range of the Gas Analyzer

The assessment of fire hazards and toxicity of PUR and PVC polymers using the Omega 5 gas analyzer was conducted in several stages. The first stage involved selecting an appropriate sample mass and the spectral range of gas spectra that would enable qualitative and quantitative identification of gases generated during thermal decomposition. In the next stage, the recorded spectra were converted to ppm values using computer software, followed by conversion to mg/m^3^. The final step included compiling the selected measurement conditions for the analysis of gas emissions from PUR and PVC, converting their spectra, and estimating toxicometric indices.

**Figure 3:**
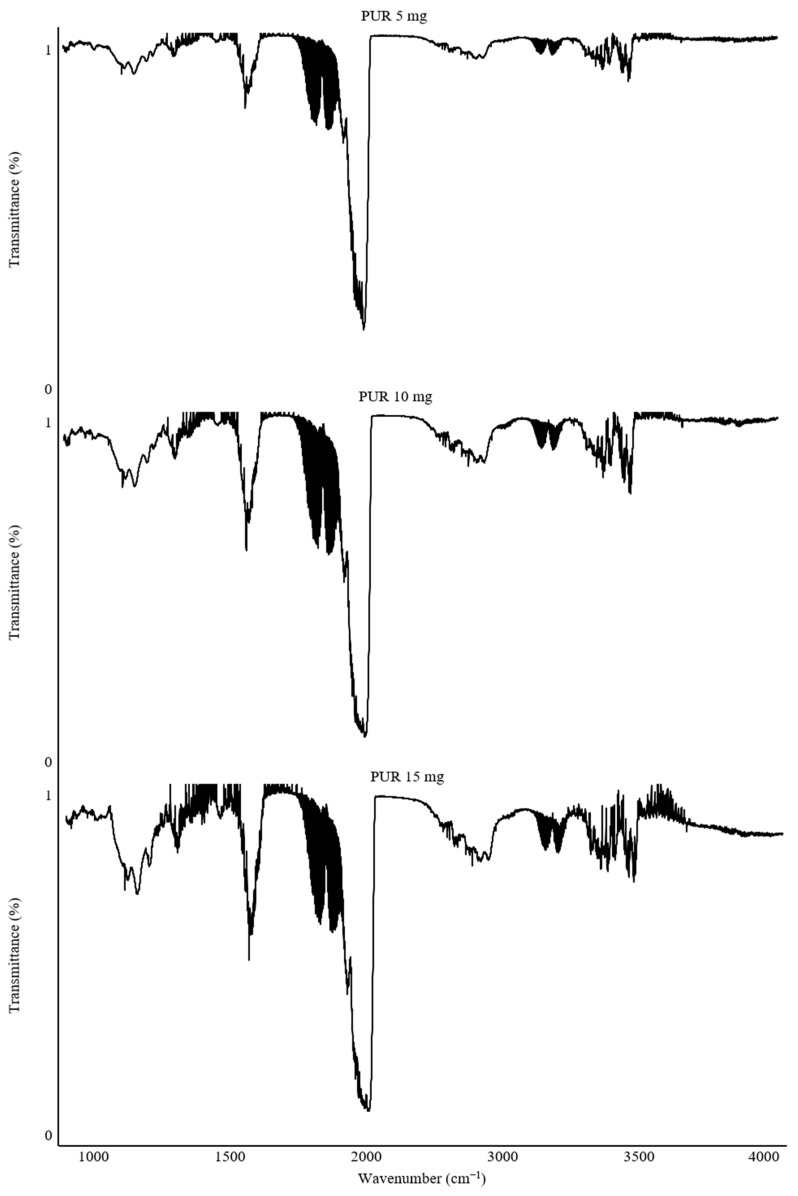
FT-IR spectra recorded during combustion of PUR samples (5 mg, 10 mg, and 15 mg) at 550 °C.

Calibration of the combusted sample mass was a critical step to ensure adequate measurement resolution. This process was fundamental for the precise determination of gas concentrations and for comparing the toxicity of the studied polymers. For this purpose, the results of spectra recorded at 550 °C for PUR samples with masses of 5 mg, 10 mg, and 15 mg were compared (Figure 3).

The recorded FTIR spectra demonstrate differences depending on the combusted mass of the PUR material. The 5 mg sample exhibited the lowest intensity of the recorded bands. The spectra for 10 mg and 15 mg PUR samples showed comparable band intensities. However, with increasing intensity, there was a noticeable rise in interference from other gas-phase combustion products related to the larger sample mass. To mitigate this issue, an alternative wavenumber range of the gas spectra can be adopted, or a smaller sample mass can be analyzed.

**Figure 4:**
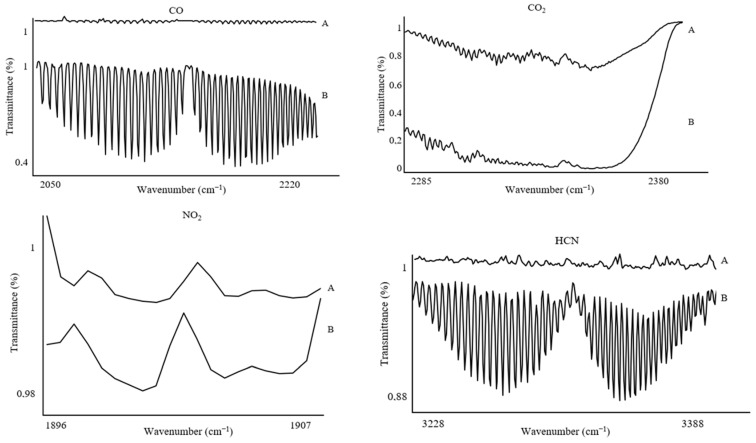
Spectral ranges of gases used during the combustion of PUR (10 mg).

The spectral ranges selected for calculating concentrations and detecting gases, applied for each sample mass, were as follows: CO (2050–2220 cm^−1^), CO_2_ (2285–2380 cm^−1^), HCN (3228–3388 cm^−1^), HCl (2744–2850 cm^−1^), NO (1896–1907 cm^−1^), NO_2_ (1579–1607 cm^−1^), NH_3_ (925–966 cm^−1^), HBr (2585–2652 cm^−1^), HF (3997–4114 cm^−1^), and SO_2_ (1341–1354 cm^−1^) (Figure 4). It is worth noting that these gases are typically characterized by broad absorption bands. However, to accurately calculate gas concentrations, it was necessary to use spectral ranges with a minimum wavenumber span of 10 cm^−1^. This requirement arises from the presence of various by-products of thermal decomposition, which can cause interference and hinder the analysis of the spectrum across its full range. Restricting the analysis to narrower bands allows for precise determination of the concentrations of individual gases.

The Opus GA software enabled the conversion of gas spectra and recorded the maximum gas concentrations. The maximum concentrations, expressed in ppm (Table 1), were used to calculate the percentage difference index (Dif%), which represents the theoretical impact of material quantity on the resolution and accuracy of the measurement (Table 2).

The percentage difference index between the maximum concentrations (Dif%) for the analyzed gases was calculated using the following formula:

(1) $$Dif\%\left(1\right)=|\frac{{PUR}_{5mg}{Gas}_{n}\left[ppm\right]\times 100\%}{{PUR}_{10}{Gas}_{n}\left[ppm\right]}-100\%|$$

(2) $$Dif\%\left(2\right)=|\frac{{PUR}_{10mg}{Gas}_{n}\left[ppm\right]\times 100\%}{{PUR}_{15}{Gas}_{n}\left[ppm\right]}-100\%|$$

Calculations of the percentage difference index (Dif%) indicate that measurements of maximum gas emissions for PUR samples with masses of 10 and 15 mg exhibit the smallest percentage differences between the maximum concentrations of individual gases (Dif% (2)) (Table 1 and Table 2).

In contrast, for PUR samples with masses of 5 and 10 mg, the percentage difference index (Dif% (1)) reaches values two to three times higher compared to Dif% (2), indicating a significant discrepancy between the obtained measurements. The results for Dif% (1) and Dif% (2) suggest that the mass range of 10 mg to 15 mg is optimal for achieving measurements with high accuracy and mutual comparability (Table 1 and Table 2).

To further verify the stability and precision of measurements for different sample masses, in the next stage, the statistical coefficient of variation (CV) was analyzed. The CV expresses the degree of data dispersion relative to the mean and is calculated using the following formula:

(3) $$CV=\frac{\sigma }{\mu }\times 100\%$$

(4) $$\mu =\frac{\sum_{i=1}^{n}x_{i}}{n}$$

(5) $$\sigma =\sqrt{\frac{\sum_{i=0}^{n}{\left(x_{i}-\mu \right)}^{2}}{n-1}}$$

where

CV—coefficient of variation;
(*μ*)—arithmetic mean;
(*σ*)—standard deviation.

Typically, for accurate parameter estimation, the value n − 1 is used. However, in this case, to compare a small sample (n = 3) and a sample reduced by one variable (n = 3 − 1), the following formula was applied:

(6) $$\sigma =\sqrt{\frac{\sum_{i=0}^{n}{\left(x_{i}-\mu \right)}^{2}}{n}}$$

where

*σ*—standard deviation;
x_i_—i-th measurement in a given series;
*μ*—mean of the measurements;
n—number of measurements.

The coefficient of variation (CV) is a valuable tool for evaluating the variability of measurements and their accuracy depending on the sample mass analyzed. A coefficient of variation with the following ranges is interpreted as follows: CV < 10% indicates low variability and high repeatability of measurements; CV between 10% and 20% indicates moderate variability, and CV > 20% indicates high variability and potential issues with repeatability or accuracy.

Analysis of the results for different masses of PUR samples shows that the relationship between samples of 5 mg and 10 mg mass is characterized by high variability (CV > 20%) (Table 3). This indicates that measurements for the 5 mg sample may be inaccurate and poorly repeatable, suggesting that this mass is insufficient for precise gas emission analyses.

For samples with masses of 10 mg and 15 mg, low variability (CV < 10%) was observed for CO_2_ and CO gas emissions, indicating high repeatability and accuracy of measurements within this mass range. However, for HCN and NO_2_ gases, the CV index indicates moderate or high variability (CV > 10%). This is most likely due to the challenges in detecting gases present in low concentrations, which are more susceptible to interference compared to other gases with overlapping absorption bands in the same FTIR spectral range (Table 3).

**Figure 5:**
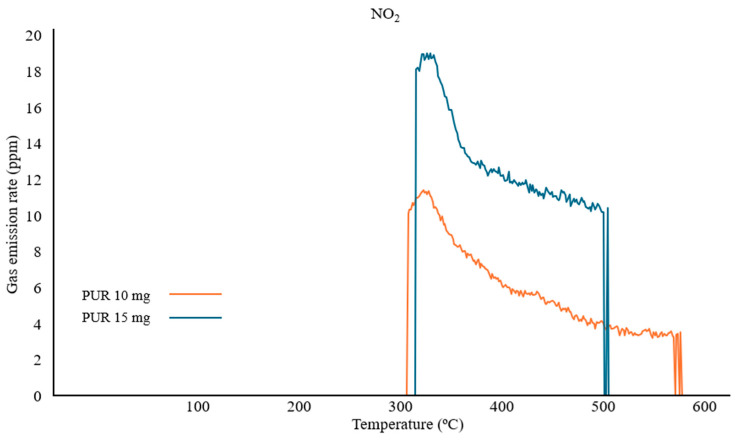
Real-time NO_2_ emission for PUR samples of 10 mg and 15 mg.

To verify the increased CV index for NO_2_ measurements between samples of 10 mg and 15 mg, a real-time emission analysis was compared. It was observed that the maximum concentration and the trend of the recorded NO_2_ gas curve for the 10 mg sample were lower than those for the 15 mg sample. However, the recorded peaks for PUR 10 mg are characterized by higher sensitivity, as evidenced by earlier gas detection and the absence of interrupted readings during thermal decomposition after 500 °C (Figure 5).

Considering the calculations and the recorded real-time emissions, it can be concluded that the optimal sample mass required to achieve adequate resolution and sensitivity during measurements with the Omega 5 gas analyzer coupled with a thermogravimetric analyzer (TG) is at least 10 mg.

### 3.2. Toxicity Analysis of PUR and PVC

The developed research methodology is essential for accurately assessing the toxicity of polymers and, consequently, for correctly determining toxicometric indices. Proper calculation of the WLC_50_ index, which represents the toxicity of gases released during thermal decomposition, is a critical part of fire hazard assessment. It allows for the estimation of material toxicity under conditions that account for the actual proportions of degradation products released. To calculate WLC_50_, reference must be made to the standardized index used in toxicological studies, LC_50_ (the threshold concentration of a given degradation product). LC_50_ is typically a measure of the toxicity of a chemical substance and represents the concentration that causes the death of 50% of a population within a 30 min exposure period (Table 4).

Using the coupled TG-gas analyzer system, two toxicity indices were developed: the Toxicity Index of Gaseous Concentration (IT_GC_) and the Toxicity Index of Partial Concentration (IT_PC_).

To calculate the above-defined indices (IT_GC_, IT_PC_), which allow for determining the material’s toxicity based on a small experimental model, the maximum concentration value of gaseous degradation products recorded during sample combustion in the range of 30–600 °C was used (Table 5).

The conversion of gas emission obtained in ppm to concentration in mg/m^3^ is expressed by the following formula:

(7) $$C=\frac{ppm\times M}{22,414}$$

where

C—concentration in mg/m^3^;
M—molar mass in g/mol;
22,414—constant (dm^3^/mol).

The calculation of the partial toxicity index (IT_PC_) based on Table 6 is expressed by the following formula:

(8) $${IT}_{PC}=\frac{C}{{LC}_{50}}$$

The IT_GC_ index value is calculated as the sum of all recorded gases (sum of IT_PC_). By analyzing Table 7, it can be observed that comparing emissions for individual gases reveals trends in the increase or decrease in the toxicity of specific gases. For instance, comparing CO emissions between PUR and PVC, it is evident that PUR is more toxic. This method of comparison can be particularly practical when assessing changes in the emission of specific gases between modified composites of a single polymer. When comparing different polymers, information about individual gases is important, but the cumulative IT_GC_ value is equally valuable. For PUR, the IT_GC_ value is 2.17, while for PVC, it is 13.07. Since the TG-gas analyzer method is relatively new and lacks standardized toxicity assessment ranges, it is not yet possible to classify toxicity ranges definitively. Toxicity classification ranges could assign classes to materials, for example: IT_GC_ > 1—toxic material; IT_GC_ < 1 < 0.7—moderately toxic material; IT_GC_ < 0.6—non-toxic material. To develop such a classification model, a standardization analysis should be conducted using certified reference materials of known toxicity under other standards. In this case, evaluating these polymers as toxic is valid but may be subject to misinterpretation.

One of the most effective ways to assess toxicity is by comparing the variability of the IT_PC_ index relative to a reference material, such as a pure base polymer or a material used in industrial practice. Such comparisons allow for the determination of the relative toxicity of the tested materials and facilitate the classification of their toxicological properties. Including IT_PC_ variability in the analysis can also help in better understanding the influence of various processing parameters and modifications on the toxicity of emitted gases. However, there are currently no reference material values obtained using the Omega 5 gas analyzer.

### 3.3. Analysis of FTIR Spectrum Components

**Figure 6:**
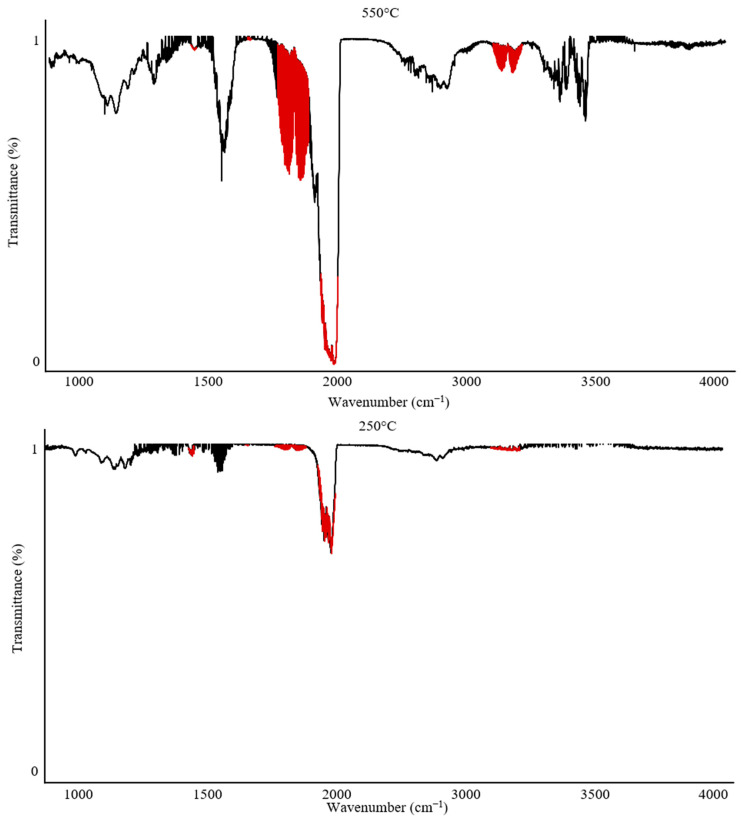
TG-FTIR spectra for PUR at 250 °C and 550 °C. The gases highlighted in red.

**Figure 7:**
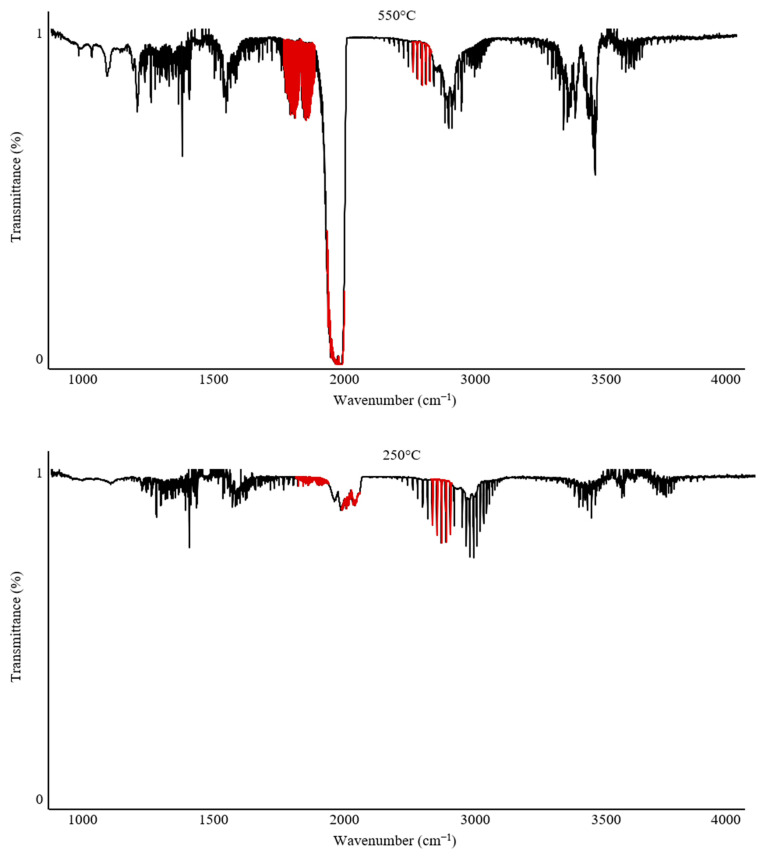
TG-FTIR spectra for PVC at 250 °C and 550 °C. The gases highlighted in red.

The Omega 5 gas analyzer with an FTIR spectrophotometer enables not only complex quantitative analyses but can also be used for qualitative and qualitative–quantitative analysis of gaseous degradation products. Figure 6 and Figure 7 illustrate the variability in band intensities depending on the temperature. Additionally, the identified gases described in Section 2.1 are marked in red. The FTIR spectrum for PUR at 250 °C recorded only CO_2_ emission at 12 ppm. In contrast, the analysis of PUR at 550 °C recorded all gases and their peak emission intensities: CO—840 ppm; CO_2_—900 ppm; HCN—62 ppm; NO_2_—4 ppm.

The analysis of PVC under the same conditions showed that at 250 °C, emissions of CO—10 ppm and HCl—300 ppm were recorded, with the temperature range of 250–300 °C being the peak range for HCl emission during the thermal decomposition of PVC. At 550 °C, the peak emission values recorded were CO—300 ppm, CO_2_—1750 ppm, and HCl—100 ppm.

The analysis of individual spectra at various temperatures can provide valuable information when characterizing the onset of gas emissions and can also complement the characterization of processes occurring during the thermal decomposition of polymers.

The recorded FTIR spectra for PUR clearly indicate that the main emission of all toxic combustion products begins after 250 °C, reaching its maximum values around 500–550 °C. In contrast, PVC exhibits a characteristic peak emission of HCl after 250 °C, while the highest concentrations of other combustion products are observed around 550 °C. This suggests that these materials are significantly different in their mechanisms of toxic substance emissions. Comparing PUR and PVC, it can be concluded that PVC poses a significantly greater hazard during the early phase of a firehazard, which is associated with flame spread and the initial stage of smoke production. This result confirms the toxicity of both materials. However, this comparative analysis suggests that PVC may present a severe health risk due to early exposure to HCl during the initial stages of a fire hazard.

## 4. Conclusions

The article presents the development of a methodology for analyzing gases from PUR and PVC using a coupled TG-gas analyzer system. In the initial stage of the study, it was demonstrated that analyses conducted with sample masses ranging from 10 mg to 15 mg exhibit high repeatability and adequate resolution of FTIR spectra, enabling the identification of essential gases such as CO_2_, CO, HCN, NO_2_, and HCl. Based on standardized methods for determining material toxicity, a proprietary toxicity index was developed. Subsequently, the study showcased the potential for applying the selected analytical conditions to characterize toxic products formed during the thermal decomposition of polymers, as well as methods for characterizing and comparing the results.

Ultimately, the selection of an appropriate sample scale depends on the specific characteristics of the tested material and the purpose of the analysis. In cases where the identification of all components is crucial, larger samples may be necessary, even at the expense of sensitivity. Conversely, for analyses requiring high precision in determining toxic gases, a smaller sample scale may be more suitable. Each of the described approaches to interpreting results has its advantages and limitations, highlighting the need for further research and development of the TG-gas analyzer method.

The innovative method for determining toxicity indices in the form of IT_PC_ and IT_GC_, presented in this article, has some limitation related to sample scale. The analysis is conducted on a small sample that can be applied to the TG device carrier (2 g), which is responsible for the thermal decomposition of the sample. This constraint may lead to false non-detection of certain toxic gases due to the insufficient amount of filler in the polymer material as a result of the limited sample mass used in the analysis. However, this limitation also presents an advantage, as the small amount of combusted material helps to reduce the formation of harmful residues and gaseous combustion products that pose a risk to personnel, while still maintaining adequate resolution of the recorded results.

Despite the high detection sensitivity (ppm), this method is not a reference standardization for materials across various industrial sectors. This is because the dedicated gas analyzer system described in this manuscript, equipped with an FTIR spectrometer coupled with a TG analyzer, does not yet have a reference material database that could serve as a standardized point of comparison. Thus, an unambiguous determination of whether a material is toxic or not will require the development of reference material databases, which will then be compared with existing regulatory standards. The next phase of research and a key objective for advancing innovative gas detection methods will involve expanding the range of analyzed gases for new or different polymeric materials using the IT_PC_ and IT_GC_ indices developed in this study.

## Figures and Tables

**Table 1:** Maximum gas emissions in ppm. The standard deviation (SD) was calculated for n = 3.

Sample/Gas	CO_2_	CO	HCN	NO_2_
PUR 5 mg	407 (SD 1.21)	366 (SD 0.11)	33 (SD 0.44)	8 (SD 0.1)
PUR 10 mg	983 (SD 6.05)	892 (SD 1.20)	65 (SD 0.21)	11 (SD 0.17)
PUR 15 mg	1119 (SD 6.08)	876 (SD 2.25)	90 (SD 0.75)	19 (SD 0.20)

**Table 2:** Percentage difference index (Dif%).

	CO_2_	CO	HCN	NO_2_
Dif%(1)	58.6	59	49.2	27.3
Dif%(2)	12.2	1.8	27.8	42.1

**Table 3:** Coefficient of variation (CV) values.

	CO_2_	CO	HCN	NO_2_
The relationship between PUR 5 mg, 10 mg, and 15 mg				
**CV**	34.3	36.9	37.2	34.2
The relationship between PUR 5 mg and 10 mg				
**CV**	41.8	41.5	32.8	14.8
The relationship between PUR 10 mg and 15 mg				
**CV**	0.9	6.5	16.0	25.0

**Table 4:** Reference LC_50_ values according to NIOSH.

LC_50_	CO_2_	CO	HCN	NO_X_	HCl
ppm	72,000	1380	55	38	75
mg/m^3 (×)^	141,372	1724	66	78	122

^(×)^ Conversion of concentration from ppm to mg/m^3^ according to formula (7).

**Table 5:** Maximum gas values (ppm).

Composite	CO_2_	CO	HCN	NO_2_	HCl
PUR	983	892	65	11	-
PVC	1839	301	-	-	1473

**Table 6:** Maximum gas values (mg/m^3^).

Composite	CO_2_	CO	HCN	NO_2_	HCl
PUR	1930	1114	79	23	-
PVC	3611	376	-	-	2397

**Table 7:** Calculated IT_PC_ values.

Composite	CO_2_	CO	HCN	NO_2_	HCl	IT_GC_
PUR	0.01	0.65	1.18	0.33	-	2.17
PVC	0.03	0.22	-	-	12.07	13.07

## Data Availability

The original contributions presented in this study are included in the article. Further inquiries can be directed to the corresponding author.
